# Supplementary material for: Vegetation structure of plantain-based agrosystems determines numerical dominance in community of ground-dwelling ants
Source: PeerJ. 2017 Nov 13;5:e3917. doi: 10.7717/peerj.3917 (PMC5689021; doi:10.7717/peerj.3917)
Supplement: Supplemental Information 2 [file peerj-05-3917-s003.pdf]

Supporting information

Table S1. Results of binomial Generalized Linear Models (using Likelihood Ratio Tests) to assess the season effect (rainy *vs.* dry) on the occurrence of each ant species/genus.

Genus/species	Chi²	p-value
Pheidole spp.	4.8132	**0.02824**
Axinidris murielae	74.941	**< 0.0001**
Paratrechina longicornis	1.9471	0.1629
Tetramorium sp.	1.123	0.2893
Monomorium spp.	0.62509	0.4292
Monomorium bicolor	16.328	**< 0.0001**
Monomorium sp. 1	0.8597	0.3538
Monomorium sp. 2	8.354	**0.003848**
Camponotus spp.	0.066346	0.7967
Camponotus acvapimensis	0.019048	0.8902
Camponotus brutus	8.0968	**0.004434**
Camponotus sp. 1	8.354	**0.00384**
Camponotus sp. 2	4.1679	**0.0412**
Odontomachus troglodytes	0.16217	0.6872

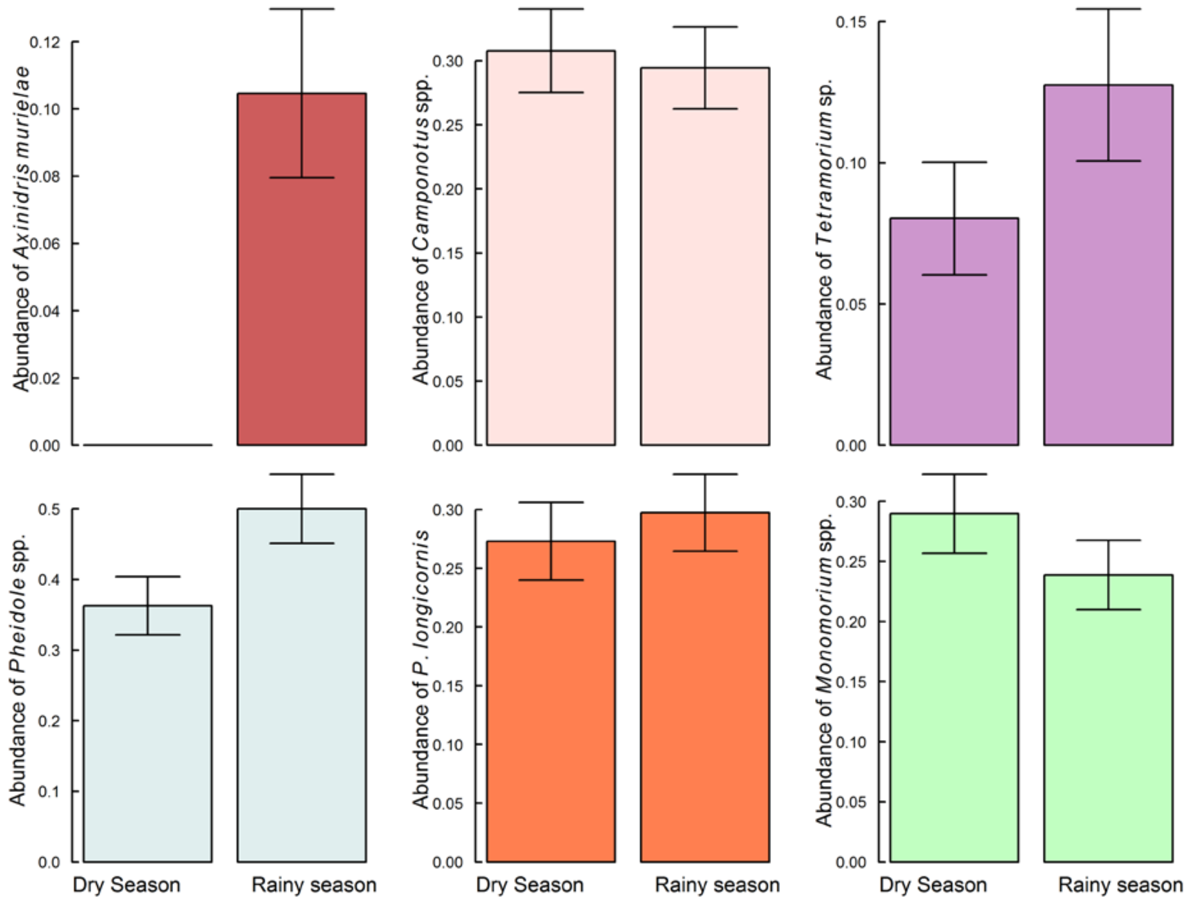

Figure S1. Log of the mean of abundance at baits of each ant taxon in the rainy and dry seasons. Error bars are 95% confidence intervals.

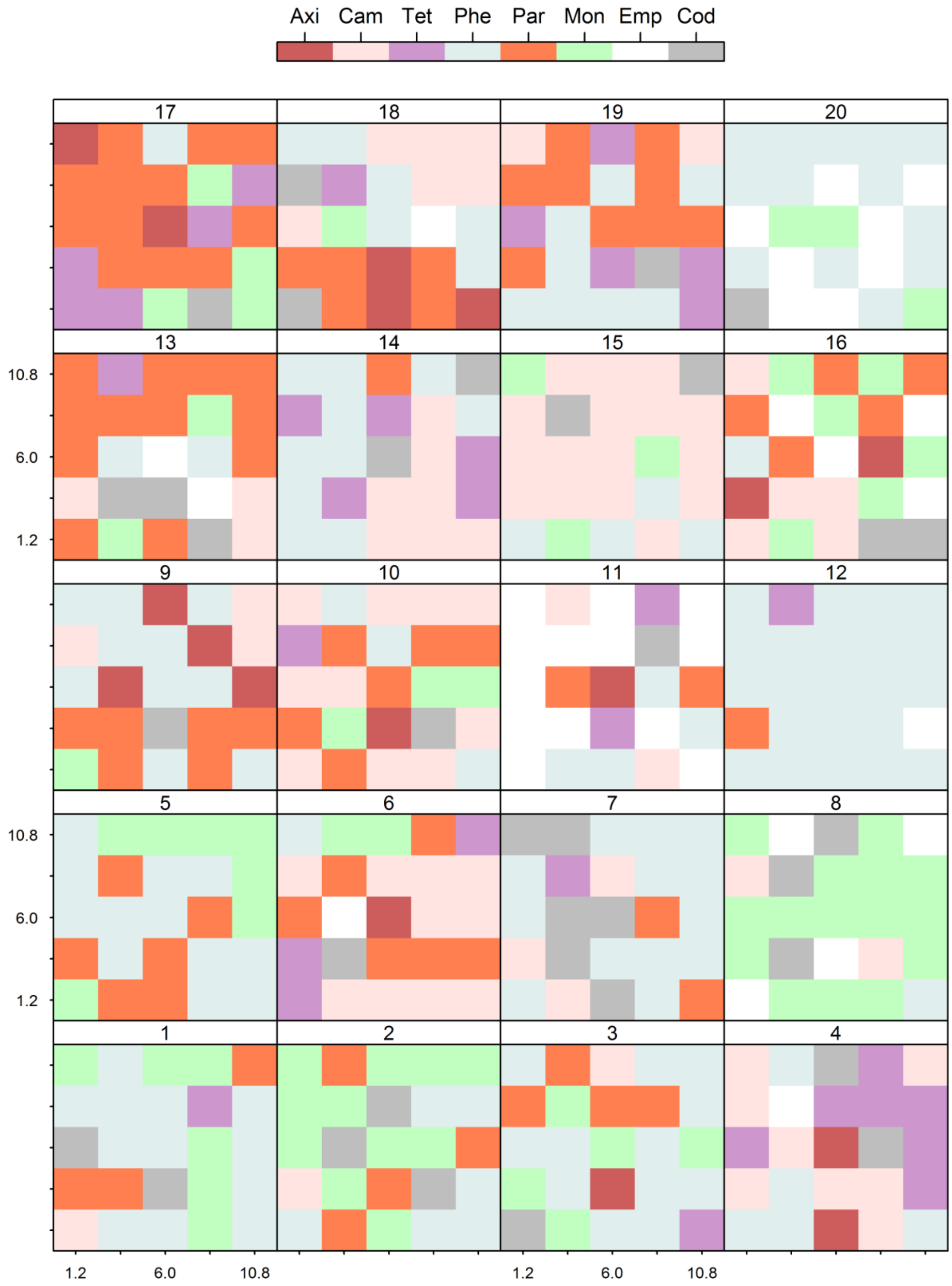

Figure S2. Numerical dominance of each ant taxon during the dry season in the 500 subplots (20 fields x 1 plot per field x 25 subplots per plot). Numbers correspond to field identification. Axin: *Axinidris murielae.*; Camp: *Camponotus* spp.; Tetra: *Tetramorium* sp.; Phei: *Pheidole* spp.; Para: *Paratrechina longicornis*; Mono: *Monomorium* spp.
